# Case Report: The carney complex led to the tragic passing of a young girl in the prime of her life

**DOI:** 10.3389/fcvm.2025.1603557

**Published:** 2025-08-25

**Authors:** Shurong Yun, Xingquan Liu, Yaxi Wang, Zhiping Liu, Jing Wu, Shasha Duan, Xiaoshan Zhang, Yilu Shi

**Affiliations:** ^1^ Department of Ultrasound, The Affiliated Hospital of Inner Mongolia Medical University, Huhhot, China; ^2^ Radiology Department, The Traditional Chinese and Mongolian Medicine Hospital of Huhhot, Huhhot, China; ^3^ Department of Cardiac Surgery, The Affiliated Hospital of Inner Mongolia Medical University, Huhhot, China; ^4^ Department of Pathology, The Affiliated Hospital of Inner Mongolia Medical University, Huhhot, China

**Keywords:** carney complex, cardiac myxoma, recurrence, *PRKAR1A*, endocrine

## Abstract

**Objective:**

We identified a novel mutation in the protein kinase cAMP-dependent type I regulatory subunit α (*PRKAR1A*) gene in a Chinese patient presenting with multiple recurrent cardiac myxomas, confirming a diagnosis of Carney complex (CNC). By reviewing the relevant literature, we aimed to enhance our understanding of this condition.

**Case presentation:**

A 12-year-old girl was referred to the Department of Cardiac Surgery at our hospital due to multiple cardiac myxomas. She had previously undergone two surgical resections of cardiac myxoma, both of which recurred shortly after the procedures. Physical examination revealed a blood pressure of 118/76 mmHg, body weight of 43 kg, height of 158 cm, and body mass index of 17.2 kg/m². No obvious skin pigmentation or nevi were observed. Whole-exome sequencing revealed a mutation in *PRKAR1A* (c.329dupC: p. A110fs), and the patient was diagnosed with CNC. During follow-up, the child succumbed to heart failure.

**Discussion:**

CNC is a rare autosomal dominant endocrine neoplasia syndrome, with approximately 53% of affected individuals having a history of cardiac myxoma. This condition should be suspected in patients initially diagnosed with multiple cardiac myxomas. Early diagnosis and treatment through multidisciplinary cooperation can improve prognosis.

## Introduction

The Carney complex (CNC) is a rare autosomal dominant multiple endocrine neoplasia syndrome characterized by mucocutaneous lentigines, cardiac myxomas, and endocrine hyperactivity (1). To date, just over 1000 related CNC cases have been reported globally (2). A mutation in the protein kinase cAMP-dependent type I regulatory subunit *α* (*PRKAR1A*) gene, located at the chromosomal level of 17q22-24 and encoding the regulatory subunit of cAMP-dependent protein kinase A (PKA), is observed in approximately 70% of CNC cases (3). Gene mutations can lead to the loss of function of the PKA regulatory subunit and unrestricted activation of the catalytic subunit, resulting in cell proliferation and tumor formation (4). Other mutated genes, including *PRKACA*, *PRKACB*, *PDE11A*, and *PDE8B*, have also been reported to be associated with CNC (5–9). CNC requires a multidisciplinary approach for diagnosis based on the patient's clinical manifestations, genetic history, molecular genetics, and personalized treatment. This article reports the case of a child initially diagnosed with multiple cardiac myxomas who underwent two thoracotomy surgeries. A novel *PRKAR1A* mutation was observed in this patient.

## Case report

A 12-year-old girl presented with lower limb edema to the Department of Cardiac Surgery at our hospital. Physical examination revealed a blood pressure of 118/76 mmHg, body weight of 43 kg, height of 158 cm, and body mass index of 17.2 kg/m². No icterus, rash, petechiae, freckles, or nevi on the skin or mucous membranes were observed. Moreover, bilateral eyelids and pitting edema of both lower limbs were also noted. She had a history of two cardiac myxoma surgeries, performed in March 2020 and June 2021. As the girl was adopted from a welfare home and had no contact with her biological parents, obtaining a detailed family history was not possible.

**Figure 1:**
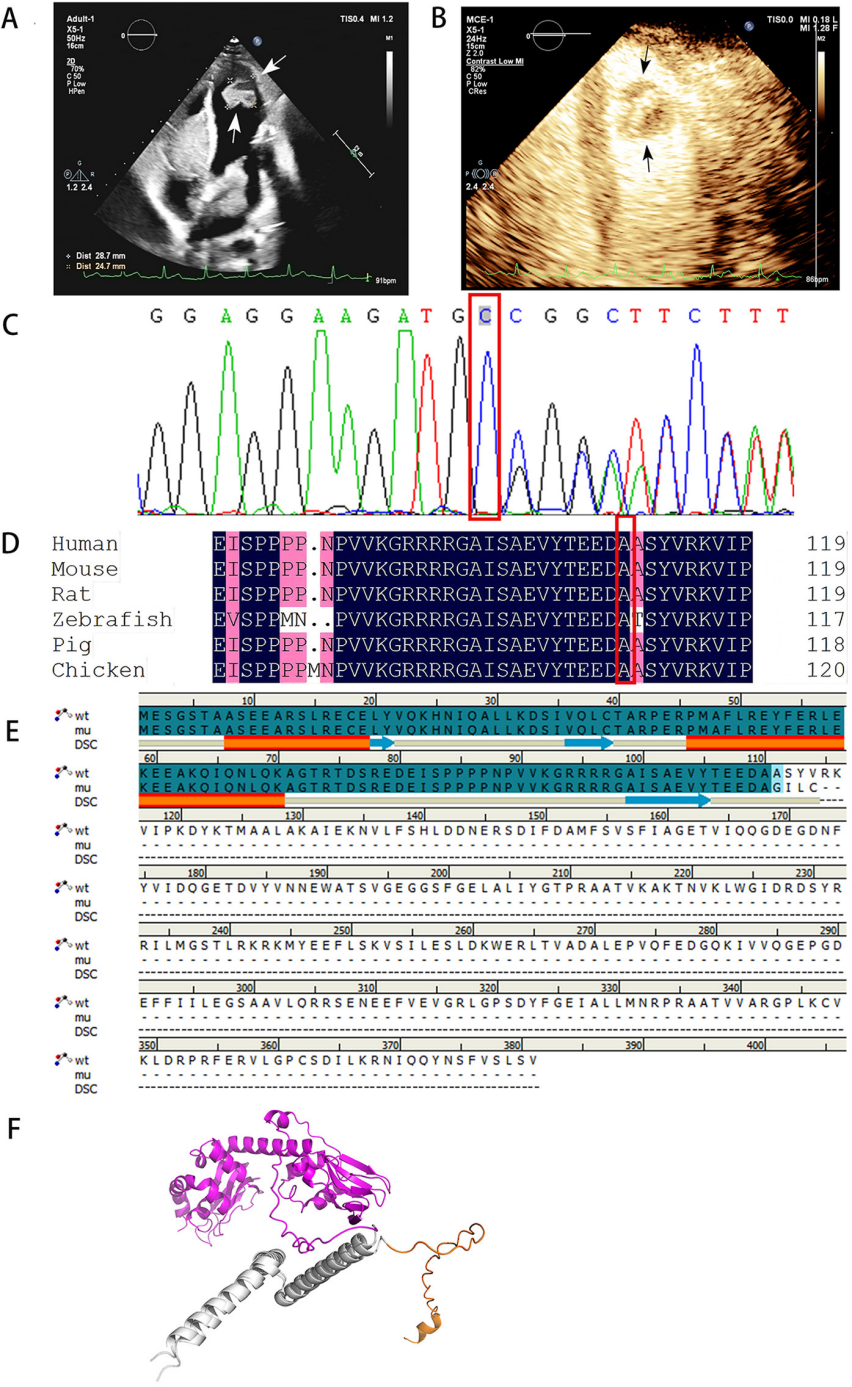
Echocardiography and sequencing results of patient case. **(A)** Echocardiography showed multiple myxomas in the left atrium, right atrium, and left ventricles, the arrows in the figure indicate the myxoma at the apex of the left ventricle. **(B)** The result of left heart contrast-enhanced ultrasound. The arrow in the figure indicates that the perfusion intensity of the myxoma at the apex of the left ventricle is close to that of the myocardium. **(C)** Sanger sequencing of PRKAR1A (c.329dupC:p.A110fs) mutation. **(D)** The P. A110fs mutation site is highly conserved. **(E)** The sequence alignment diagram of wild-type and mutant p.A110fs shows that the p.A110fs mutation terminates translation at position 114. The first 110 segments of p.A110fs and wild-type both have two α-helices and three β-sheets. **(F)** The first 70 amino acids of the wild type and the p.A110fs mutant have a good superimposition. The orange color represents the 70–114 domain of the p.A110fs mutant, while the purple color represents the 70–381 protein domain of the wild type. Through comparison, it was found that the p.A110fs mutant mainly lacks 267 amino acids, mainly consisting of 8 β-sheets and 8 α-helices.

Echocardiography revealed multiple solid masses in the atrioventricular cavity, with the largest mass measuring 61 × 35 mm, and a left ventricular ejection fraction of 54% (Figure 1A). Contrast echocardiography demonstrated perfusion of the contrast agent within the tumor (Figure 1B). Color Doppler ultrasound of the adrenal glands, thyroid gland, and breasts revealed no abnormalities (Table 1).

**Table 1:** Ultrasound data of patients at different onset times.

Time	LA-ap (mm)	LVEDD (mm)	RA-t (mm)	RV-m (mm)	LVEF (%)	Mitral valve involvement	Tricuspid valve involvement	Tumor size (mm)
March 2020	37	43	37	36	62	Y	N	LV:39*29 LA:52*29
June 2021	35	40	35	35	61	Y	N	LV:21*21 LA:35*17
June 2023	36	40	36	35	58	Y	Y	LV:28*24 LA:42*26 RA:52*34
August 2024	42	41	40	40	54	Y	Y	LV:35*35 LA:53*34 RA:61*35

LA-ap, left atrial anteroposterior diameter; LVEDD, left ventricular end-diastolic diameter; RA-t, right atrial transverse diameter; RV-m, right ventricular mid-cavity transverse diameter; LVEF, left ventricular ejection fraction.

With the consent of her guardian, whole exome sequencing was performed on the peripheral blood samples. The sequencing results revealed a mutation in *PRKAR1A* (c.329dupC:p.A110fs), with the mutation site verified using Sanger sequencing (Figure 1C). This mutation site is highly conserved (Figure 1D). The diagnosis was made according to the Stratakis CA-Carney complex diagnostic criteria (10). Although the child presented solely with cardiac myxomas, the identification of *PRKAR1A* gene mutations confirmed the diagnosis of CNC.

**Figure 2:**
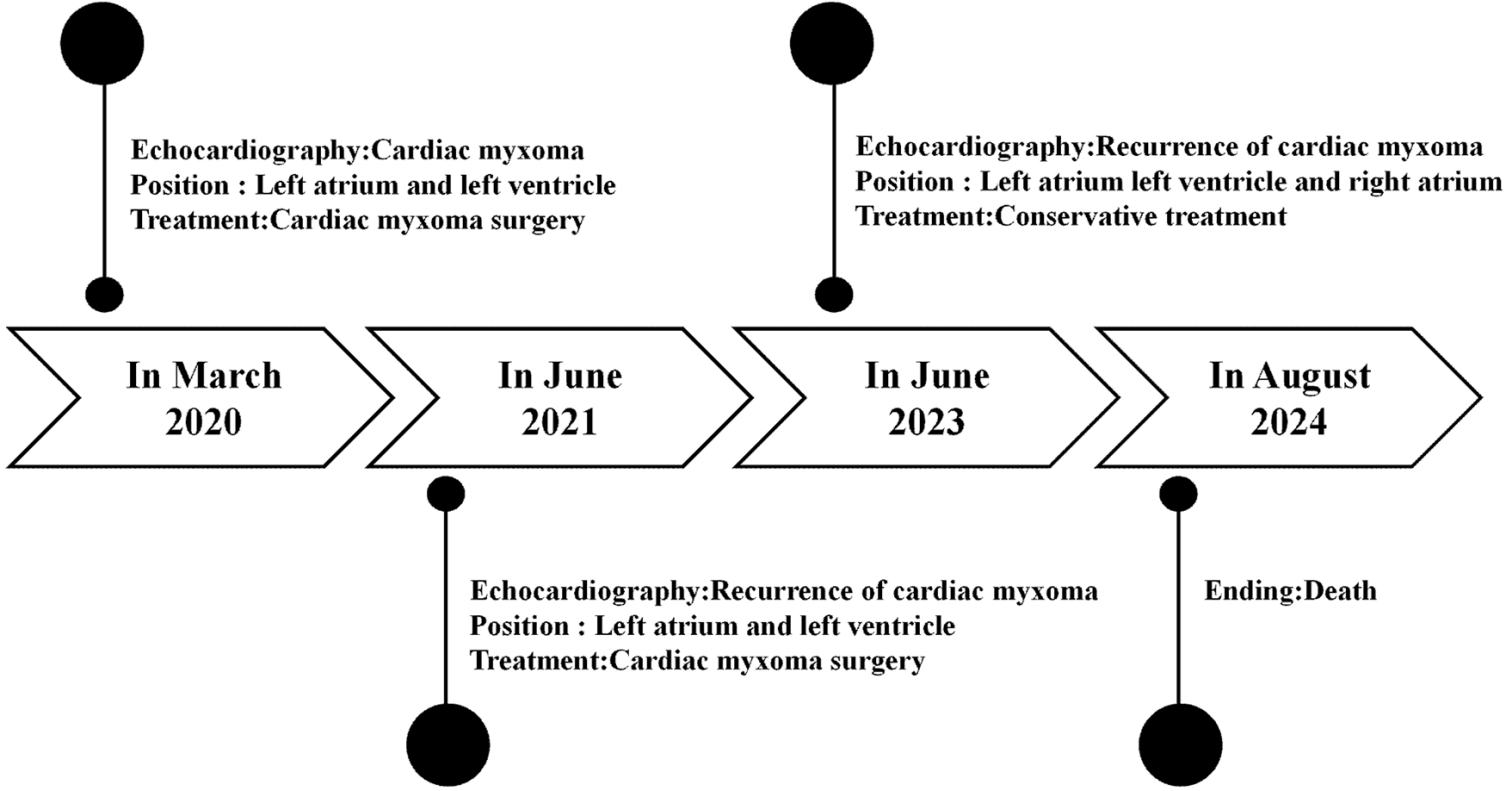
Case report of follow-up time and the corresponding examination and treatment results.

The recurrence occurred 21 months after the second surgery. The tumor had increased in size compared to prior assessments, and symptoms indicative of global heart failure had already manifested. Considering the patient's genetic background, symptomatic treatment and heart transplantation were recommended. Unfortunately, the patient died while waiting for a donor (Figure 2).

## Discussion

This study describes the case of a child with multiple cardiac myxomas. Physical examination revealed only lower limb swelling, which might have been caused by large cardiac myxomas obstructing venous return. The child did not exhibit any manifestations of Cushing syndrome, such as obesity, moon face, or a buffalo hump. Additionally, no obvious skin pigmentation or nevi were observed. Therefore, endocrinological screening was not considered, which may have contributed to the delayed diagnosis. In this case, we discovered that the child had no biological relationship with her parents, which disrupted the diagnostic process for CNC from a family history perspective. Consequently, the child experienced recurrent cycles of myxoma resection over 4 years. Due to the large number and size of recurrent myxomas and the burden of multiple thoracotomy surgeries, the patient and her family declined further surgical intervention, and she eventually died of heart failure. Therefore, clinicians are recommended to maintain heightened vigilance for the risk of recurrence in young patients presenting with multiple cardiac myxomas. Endocrinological screening should be conducted to confirm the diagnosis, even in the absence of characteristic clinical manifestations.

Currently, at least 130 mutation sites in the *PRKAR1A* gene related to CNC have been reported, including nonsense or missense mutations, short frameshift insertions or deletions, and rare large fragment deletions, all exhibiting nearly complete penetrance (1). The mutation identified in this case, c.329dupC: p.A110fs, is predicted to be pathogenic according to the American College of Medical Genetics and Genomics and has not been reported in previous studies, representing a novel pathogenic mutation. This frameshift mutation involves the insertion of a cytosine at nucleotide position 329, resulting in a shift of the reading frame beginning at the alanine residue at the 110th position. The stop codon appears in advance at position 114, generating a truncated protein, with the structural and functional domains at the C-terminal completely lost, leading to dysfunction of the encoded RIa protein (Figures 1E,F).

The incidence of cardiac myxoma is approximately 0.001‰, which is rare in the general population, but occurs in 20%–40% of patients with CNC (11). Cardiac myxoma is the primary cause of mortality and a significant contributor to morbidity in patients with CNCs, predominantly due to thrombosis or hemodynamic abnormalities caused by the tumor. Approximately 60% of patients experience recurrence of cardiac myxoma, with the first recurrence occurring at an average of 4.2 ± 3.5 years; females constitute over 70% of these cases (12). The high recurrence rate may be attributed to excessive growth hormone secretion (12, 13). Some researchers have proposed that the prophylactic reduction of growth hormone levels in patients with CNC may reduce the possibility of cardiac myxoma formation and/or recurrence (13). However, owing to the unclear disease-related phenotypes presented by the child in this study, no endocrinology laboratory tests were conducted, resulting in missed opportunities for early intervention. This may account for the multiple recurrences of cardiac myxomas in this child over a short period. Additionally, some scholars suggest that cardiac myxomas often exhibit invasive growth patterns. If only the tumor is resected during the procedure without thoroughly removing the attached intimal tissue, the residual cells may persist and regenerate, potentially leading to myxoma recurrence (14).

Herein, we report the case of a minor girl. Multiple recurrences of cardiac myxoma led us to suspect the presence of CNC. Although the patient had no obvious skin manifestations, the diagnosis was confirmed by gene sequencing. Therefore, CNCs should be suspected in patients who are initially diagnosed with cardiac myxomas. Clinically, examinations of multiple organs and sites should be performed to facilitate early diagnosis, thereby optimizing clinical decision-making.

## Data Availability

The original contributions presented in the study are included in the article/Supplementary Material, further inquiries can be directed to the corresponding author.
